# Impact of Different Trace Elements on the Growth and Proteome of Two Strains of *Granulicella*, Class “Acidobacteriia”

**DOI:** 10.3389/fmicb.2020.01227

**Published:** 2020-06-18

**Authors:** Ohana Y. A. Costa, Chidinma Oguejiofor, Daniela Zühlke, Cristine C. Barreto, Christine Wünsche, Katharina Riedel, Eiko E. Kuramae

**Affiliations:** ^1^Department of Microbial Ecology, Netherlands Institute of Ecology (NIOO-KNAW), Wageningen, Netherlands; ^2^Institute of Biology Leiden, Leiden University, Leiden, Netherlands; ^3^Department of Soil Science and Meteorology, Michael Okpara University of Agriculture, Umudike, Nigeria; ^4^Institute of Microbiology, University of Greifswald, Greifswald, Germany; ^5^Genomic Sciences and Biotechnology Program, Catholic University of Brasilia, Distrito Federal, Brazil; ^6^Ecology and Biodiversity, Institute of Environmental Biology, Utrecht University, Utrecht, Netherlands

**Keywords:** *Acidobacteria*, *Granulicella*, genome, proteome, manganese, metabolism

## Abstract

*Acidobacteria* represents one of the most dominant bacterial groups across diverse ecosystems. However, insight into their ecology and physiology has been hampered by difficulties in cultivating members of this phylum. Previous cultivation efforts have suggested an important role of trace elements for the proliferation of *Acidobacteria*, however, the impact of these metals on their growth and metabolism is not known. In order to gain insight into this relationship, we evaluated the effect of trace element solution SL10 on the growth of two strains (5B5 and WH15) of *Acidobacteria* belonging to the genus *Granulicella* and studied the proteomic responses to manganese (Mn). *Granulicella* species had highest growth with the addition of Mn, as well as higher tolerance to this metal compared to seven other metal salts. Variations in tolerance to metal salt concentrations suggests that *Granulicella* sp. strains possess different mechanisms to deal with metal ion homeostasis and stress. Furthermore, *Granulicella* sp. 5B5 might be more adapted to survive in an environment with higher concentration of several metal ions when compared to *Granulicella* sp. WH15. The proteomic profiles of both strains indicated that Mn was more important in enhancing enzymatic activity than to protein expression regulation. In the genomic analyses, we did not find the most common transcriptional regulation of Mn homeostasis, but we found candidate transporters that could be potentially involved in Mn homeostasis for *Granulicella* species. The presence of such transporters might be involved in tolerance to higher Mn concentrations, improving the adaptability of bacteria to metal enriched environments, such as the decaying wood-rich Mn environment from which these two *Granulicella* strains were isolated.

## Introduction

Despite being widespread and dominant in soil ecosystems (Kuramae et al., 2012; Navarrete et al., 2013b; Pereira de Castro et al., 2016), the phylum *Acidobacteria* has a low number of cultivated representatives, due to difficulties in isolation and propagation under laboratory conditions (Dedysh and Yilmaz, 2018). Most *Acidobacteria* isolates are slow growers and can take weeks to months to develop colonies (Eichorst et al., 2011; de Castro et al., 2013). Recently, changes in traditional culture methods and application of unconventional culture media composition have increased the number of new *Acidobacteria* isolates considerably. Currently, 62 species with validated names have been described (NCBI Resource Coordinators, 2016), while in 2011 only 14 species had been isolated and characterized (de Castro, 2011). Modifications in culture media and cultivation conditions, such as low concentration of nutrients (Janssen et al., 2002; Stevenson et al., 2004), higher CO_2_ concentrations (Stevenson et al., 2004), unusual or complex polysaccharides as carbon sources (Pankratov et al., 2008; Eichorst et al., 2011), longer incubation periods (de Castro et al., 2013), addition of humic acids and quorum-sensing molecules (Stevenson et al., 2004), employment of soil solution equivalents and inhibitors for unwanted microorganisms (de Castro et al., 2013; Foesel et al., 2013), are strategies that have been applied for the enrichment and isolation of new *Acidobacteria* species.

Once the isolates are obtained, better cell proliferation can be achieved with richer culture media, containing higher concentrations of nutrients (de Castro et al., 2013). For instance, trace elements can be used to improve microbial growth and gain of biomass under laboratory conditions, even though the specific requirements among strains and species are variable (Banerjee et al., 2009; Merchant and Helmann, 2012). Metal ions such as Fe, Mn, Zn, and Cu are fundamental for microbial metabolism, being required at low concentrations (Abbas and Edwards, 1990). They play an important role in biological processes, acting as co-factors of enzymes (Wintsche et al., 2016), activating metalloregulators and trace element dependent proteins (Hantke, 2001; Zhang et al., 2009), forming functional complexes with secondary metabolites (Morgenstern et al., 2015; Locatelli et al., 2016) and promoting the detoxification of reactive oxygen species (ROS) (Kehres and Maguire, 2003; Locatelli et al., 2016).

Although some culture media used for *Acidobacteria* growth and isolation are supplemented with trace elements (de Castro et al., 2013; Navarrete et al., 2013a), the impact of these metals on their growth and metabolism is not yet known. Though metal ions are essential for many biological processes, they can be toxic at high concentrations (Puri et al., 2010). Metal ions cannot be synthesized or degraded, therefore cellular homeostasis of metals relies mostly on transport, which involves several mechanisms that sense, uptake, immobilize or pump metals out of the cell (Chandrangsu et al., 2017). In the present study, we evaluated the effect of trace elements and particularly Mn on the growth of *Granulicella* spp. WH15 and 5B5, *Acidobacteria* (class “Acidobacteriia”), derived from decaying wood (Valášková et al., 2009). We used the optimized culture medium PSYL5 (Campanharo et al., 2016) to boost the growth of the two strains, evaluated the impact of Mn through proteome studies and performed genomic analyses on both strains.

## Materials and Methods

### *Acidobacteria* Strains

Two strains of *Acidobacteria*, 5B5 and WH15 belonging to *Granulicella* genus of class “Acidobacteriia” were used in this study. Both strains belong to the culture collection of Netherlands Institute of Ecology (NIOO-KNAW), Department of Microbial Ecology. They were isolated from wood in advanced decay stage, in association with the white-rot fungus *Hypholoma fasciculare*, in Netherlands (Valášková et al., 2009). The genome of *Granulicella* sp. WH15 is deposited at NCBI with accession number CP042596 while the genome of *Granulicella* sp. 5B5 was sequenced in this study.

### Trace Elements Solution (SL10) and Individual Trace Elements Effect on Bacterial Growth

The effect of trace element solution SL10 (Widdel et al., 1983) on the growth of both bacterial strains was evaluated for two different concentrations (1 and 10 ml) of the solution per L of PSYL5 culture medium. PSYL5 medium was composed of (g/L): 1.8 KH_2_PO_4_, 0.2 MgSO_4_.7H_2_O, 30 sucrose and 1.0 yeast extract; pH was adjusted to 5.0 (Campanharo et al., 2016). Culture medium without the amendment of SL10 solution was used as a control. Seven-day-old cell suspensions of both strains were inoculated in 70 ml of culture medium to an OD_600__nm_ 0.01. The cultures were incubated under aeration for 7 days at 30°C and a constant rotation rate of 50 rpm. Every 24 h the optical density of the cultures was measured with an Eppendorf photometer at a wavelength of 600 nm (Eppendorf, Hamburg, Germany). For the evaluation of the different trace elements on the growth of both strains, individual trace element stock solutions and growth curves were executed for each metal separately, using the same growth conditions described above. The composition of the trace element solution SL10 and the final concentration of each trace element in the culture medium is shown in Supplementary Table S1. The metal salt that produced a significantly higher growth in comparison with the control was selected for further experiments. All experiments were executed in triplicates.

Statistical analysis was performed using SigmaPlot v14. Normality of the data was checked using Shapiro–Wilk test. Two-way repeated measures ANOVA was used to test the effect of SL10 solution and individual trace elements on the growth rate of *Granulicella* spp. WH15 and 5B5.

### Genome of *Granulicella* sp. 5B5

The *Granulicella* sp. 5B5 strain obtained from the collection of Netherlands Institute of Ecology (NIOO-KNAW) was grown on 1/10 TSB agar medium (Valášková et al., 2009) at pH 5.0 for 3 days at 30°C. The bacterial cells were harvested and the genomic DNA was extracted using MasterPure^TM^ DNA Purification Kit (Epicentre, Madison, WI, United States) according to manufacturer’s instructions. A total of 10 μg of DNA was sent to the Genomics Resource Center (Baltimore, MD, United States) for a single long insert library (15–20 kb), that was constructed and sequenced in one SMRTcell using the PacBio RS II (Pacific Biosciences, Inc.) sequencing platform. *De novo* assembly was performed with the help of SMRT Analysis software v2.2.0 (Pacific Biosciences) featuring HGAP 2 (Chin et al., 2013), and subsequent correction with Pilon 1.16 (Walker et al., 2014) to reveal a circular replicon: a 3,928,701 bp chromosome (G + C content 61, 1%; 58 × coverage). Automatic gene prediction and annotation was performed by using Prokka (Seemann, 2014) and RAST genome annotation server^1^ (Aziz et al., 2008). Predicted genes were mapped to Cluster of Ortholog Groups (COG) and KEGG IDs using the COG database (2014 release) (Galperin et al., 2015) and KEGG database (release 2013) (Kanehisa, 2000), using eggNOG mapper. The CAZyme contents of *Granulicella* sp. 5B5 genome were determined by identifying predicted genes containing CAZyme domains using the dbCAN2 meta server^2^ (Zhang et al., 2018), according to the CAZy (Carbohydrate-Active Enzyme) database classification (Lombard et al., 2014). Only CAZyme domains predicted by at least two of the three algorithms (DIAMOND, HMMER, and Hotpep) employed by dbCAN2 were kept. The phylogenetic relationship between *Granulicella* sp. 5B5, *Granulicella* sp. WH15, and other *Granulicella* species was analyzed based on 16S rRNA gene sequences. The dendrogram was constructed by using Maximum-Likelihood [Tamura-Nei model (Tamura and Nei, 1993), 1000 bootstraps] analysis, with software MEGA X (Kumar et al., 2018). Pairwise genome alignment was performed using the lastz v. 1.04 program (Harris, 2007). The results were visualized using AliTV v. 1.0.6. (Ankenbrand et al., 2017). Circular genome map was drawn using CGView software (Stothard and Wishart, 2004). Average Nucleotide Identity (ANI) between *Granulicella* spp. 5B5 and WH15 was calculated using the webtool ANI calculator, available at https://www.ezbiocloud.net/tools/ani (Yoon et al., 2017).

### Metal Tolerance Assays and Metal Resistance Gene Annotation

The metal tolerance of *Granulicella* spp. 5B5 and WH15 to varied metal salt concentrations was tested in solid culture medium (Abou-Shanab et al., 2007; Afzal et al., 2017) PSYL5 pH 5. Five concentrations (0.5, 1, 2, 5, and 10 mM) of nine metal salts were tested: ZnCl_2_, NiCl_2_, MnCl_2_, CoCl_2_, CuCl_2_, NaMoO_4_, AlCl_2_, CdCl_2_, and C_8_H_4_K_2_O_12_Sb_2_ (Abou-Shanab et al., 2007; Terry et al., 2015). As a control, an *Escherichia coli* DH5α strain, with known low metal tolerance was used. Six isolated colonies of each strain previously grown on PSYL5 solid medium without metal were streaked on the culture media with each different metal concentration. Growth was interpreted as positive when colony formation was similar to growth on control culture medium without the addition of metals. After 7 days of growth at 30°C, colonies were streaked on a new plate with the same metal concentration, in order to confirm growth. If colonies did not develop within 7 days, plates were incubated for extra 7 days. Colonies were restreaked three times for confirmation. When the strains were resistant to the highest concentration of metal used (10 mM), we performed additional tests using higher metal concentrations (15, 20, 25, 30, and 40 mM).

In order to identify predicted genes that could be involved in metal ion homeostasis, we searched the genomes of both strains against the experimentally confirmed and predicted BacMet databases using BacMet Scan (Pal et al., 2014) with less strict parameters (40% similarity), due to the high quantity of hypothetical proteins in the genomes of both bacteria.

### Acquisition of Cytosolic Proteome With and Without Manganese Treatment by Mass Spectrometry and Data Analysis

For the proteome analysis, we analyzed the effects of the metal salt which significantly improved the growth yield of both strains in comparison to the control without metal salt. Therefore, Mn was selected for further experiments. The growth curves of *Granulicella* spp. WH15 and 5B5 with added manganese (MnCl_2_) and controls without trace elements were repeated, using the same parameters as described in section “Trace Elements Solution (SL10) and Individual Trace Elements Effect on Bacterial Growth.” Cells were collected at day 4 of the growth curve, when the differences in the OD_600 nm_ between manganese treatment and control treatment started to be statistically significant. A total of 3 ml of bacterial cell culture per replicate (*n* = 3 for each treatment) were harvested by centrifugation at 10,015 × *g* at 4°C for 10 min. Pellets were washed twice with 1 mL of TE buffer and finally resuspended in 1 ml TE buffer. A volume of 500 μL of cell suspension was transferred into 2 mL screw cap tubes filled with 500 μL glass beads (0.1 mm in diameter; Sarstedt, Germany) and mechanically disrupted using Fastprep (MP Biomedicals) for 3 × 30 s at 6.5 m/s; with on ice incubation for 5 min between cycles. To remove cell debris and glass beads, samples were centrifuged for 10 min at 4°C at 21,885 × *g*, followed by a second centrifugation (30 min at 4°C at 21,885 × *g*) to remove insoluble and aggregated proteins. The protein extracts were kept at −20°C. Protein concentration was determined using RotiNanoquant (Carl Roth, Germany). Proteins were separated by SDS-PAGE. Protein lanes were cut into 10 equidistant pieces and in-gel digested using trypsin as described earlier (Grube et al., 2014). Tryptic peptides were separated on an EASY-nLC II coupled to an LTQ Orbitrap Velos using a non-linear binary 76 min gradient from 5 to 75% buffer B (0.1% acetic acid in acetonitrile) at a flow rate of 300 nL/min and infused into an LTQ Orbitrap Velos (Thermo Fisher Scientific, United States) mass spectrometer. Survey scans were recorded in the Orbitrap at a resolution of 60,000 in the m/z range of 300–1,700. The 20 most-intense peaks were selected for CID fragmentation in the LTQ. Dynamic exclusion of precursor ions was set to 30 s; single-charged ions and ions with unknown charge were excluded from fragmentation; internal calibration was applied (lock mass 445.120025).

For protein identification resulting MS/MS spectra were searched against a database containing protein sequences of *Granulicella* sp. strain 5B5 or *Granulicella* sp. strain WH15 and common laboratory contaminants (9,236 entries or 7,782 entries, respectively) using Sorcerer-Sequest v.27, rev. 11 (Thermo Fisher Scientific) and Scaffold v4.7 (Proteome Software, United States) as described earlier (Stopnisek et al., 2016). Relative quantification of proteins is based on normalized spectrum abundance factors (NSAF; Zhang et al., 2010).

Statistical analysis was done using MeV (Saeed et al., 2003); *t*-test was applied for proteins that were identified in at least two replicates of the respective condition. Hierarchical clustering and *t*-test of z-transformed normalized data were performed with the following parameter unequal group variances were assumed (Welch approximation), *p*-values based on all permutation with *p* = 0.01, significance determined by adjusted Bonferroni correction. Only significantly changed proteins showing at least 1.5-fold changes between conditions were considered for further analysis. Furthermore, so-called on/off proteins, that were only identified in one condition were analyzed. Functional classification of *Granulicella* sp. strain 5B5 and WH15 proteins was carried out using eggNOG mapper^3^ (Huerta-Cepas et al., 2017), COG (Galperin et al., 2015), and KEGG databases (Kanehisa, 2000). In order to identify proteins that could be involved in metal ion homeostasis, we searched proteins with significantly changed amounts against the experimentally confirmed and predicted BacMet databases using BacMet Scan (Pal et al., 2014) with less strict parameters (40% similarity), due to the high quantity of hypothetical proteins in the genomes of both bacteria. Voronoi treemaps for visualization of proteome data were generated with Paver software (Decodon GmbH, Germany).

## Results

### Trace Mineral Solution (SL10) Effects on Growth

**FIGURE 1:**
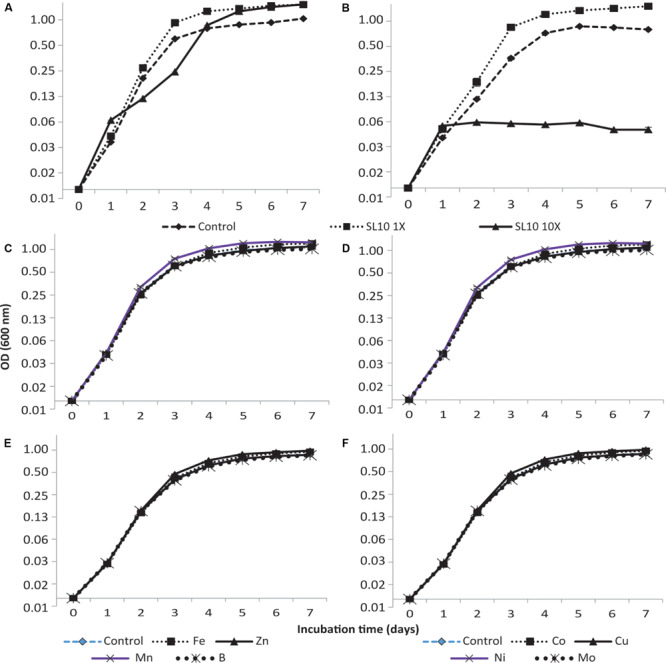
Growth curves of *Granulicella* sp. 5B5 and WH15 on PSYL5 liquid culture medium with different concentrations of trace element solution (SL10) and individual metal salts. **(A)** *Granulicella* sp. 5B5 with SL10 solution: control (no SL10), 1× SL10 and 10× SL10; **(B)** *Granulicella* sp. WH15 with SL10 solution: control (no SL10), 1× SL10 and 10× SL10; **(C)** *Granulicella* sp. 5B5 with individual metal salts: control (no metal), Fe^2+^, Zn^2+^, Mn^2+^, BO_3_^3–^; **(D)** *Granulicella* sp. WH15 with individual metal salts: control (no metal), Fe^2+^, Zn^2+^, Mn^2+^, BO_3_^3– –^; **(E)** *Granulicella* sp. 5B5 with individual metal salts: control (no metal), Co^2+^, Cu^2+^, Ni^2+^, MoO_4_^2–^; **(F)** *Granulicella* sp. WH15 with individual metal salts control (no metal), Co^2+^, Cu^2+^, Ni^2+^, MoO_4_^2–^. The error bar is the standard error of the mean (*n* = 3) and indicates differences in response variable between different treatments. Fe, FeCl_2_.4H_2_O; Zn, ZnCl_2_; Mn, MnCl_2_.4H_2_O; B, H_3_BO_3_; Co, CoCl_2_.6H_2_O; Cu, CuCl_2_.2H_2_O; Ni, NiCl_2_.6H_2_O; Mo, NaMoO_4_.2H_2_O.

The addition of trace element solution SL10 in liquid culture medium produced a significant effect (*p* < 0.001) on the growth of both strains of *Granulicella* sp. Both concentrations (1× and 10×) of trace element solution (SL10) significantly increased *Granulicella* sp. 5B5 growth, with the highest growth recorded for 1× and 10× concentrations of SL10 (Figure 1A). In addition, the cultures showed a longer lag phase for 10× concentration of SL10 (Figure 1A).

*Granulicella* sp. WH15 had a significantly higher growth rate with the addition of 1× SL10 compared to control and 10× SL10 (Figure 1B), except at day one of incubation. Differently from *Granulicella* sp. 5B5, 10× SL10 did not enhance the growth of *Granulicella* sp. WH15, having instead the opposite effect (Figure 1B).

### Effect of Individual Trace Element on Growth

Of all the trace elements, manganese (Mn) and copper (Cu) significantly increased the growth of *Granulicella* sp. 5B5 compared to control starting from day three of the incubation period until the end (*p* < 0.001) (Figure 1C). Iron (Fe) significantly increased the growth of *Granulicella* sp. 5B5 only at day 6, when compared with the control. Boron (B), zinc (Zn), cobalt (Co), nickel (Ni), and molybdate (Mo) did not have any significant effect on the growth of the strain throughout the duration of the experiment (Figures 1C,D).

Throughout the incubation period, Mn was the only trace element that significantly (*p* < 0.001) increased the growth of *Granulicella* sp. WH15 in comparison with the control with no metal (Figure 1E). Fe, Zn, Co, Cu, Ni, Mo, and Bo did not have any significant effect on the growth of *Granulicella* sp. WH15 as compared to the control (Figures 1E,F).

### *Granulicella* sp. 5B5 Genome Annotation and CAZymes

**FIGURE 2:**
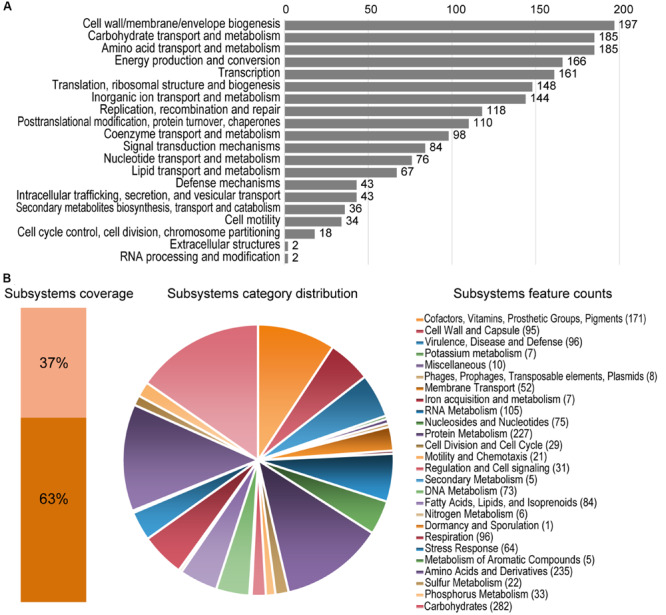
Statistics of COG and RAST subsystems annotations of *Granulicella* 5B5. **(A)** COG categories distribution, showing number of predicted genes annotated in each category. **(B)** Subsystem category distribution. The light orange bar represents the percentage of proteins that could be annotated by RAST Server and the dark orange bar represents the proteins that were not annotated. The pie chart represents the percentage of proteins annotated to each subsystem category. Following the pie chart clockwise, subsystem categories are listed in the legend from top to bottom.

The assembled genome of *Granulicella* sp. 5B5 is 3,928,701 bp, with 61.1% GC content, 3,306 proteins and only one rRNA operon. Functional annotation using COG and RAST analysis resulted in the classification of 2,615 predicted genes into 20 COG functional groups and the annotation of 1,260 predicted genes to RAST subsystems. The properties of the genomes of *Granulicella* sp. 5B5 and also *Granulicella* sp. WH15 (sequenced previously, Costa et al., 2020b) are listed in Table 1. A circular genome map of *Granulicella* sp. 5B5 is depicted in Supplementary Figure S1, together with that of strain WH15. The distribution of predicted genes into COGs/RAST functional categories for *Granulicella* sp. 5B5 genome is depicted in Figure 2. ANI (Figueras et al., 2014) between *Granulicella* spp. WH15 and 5B5 was 72.75%, showing that the strains do not belong to the same species. Comparisons among 16S sequences demonstrated that *Granulicella* sp. 5B5 is more similar to *Granulicella cerasi* and *Granulicella paludicola*, while *Granulicella* sp. WH15 is closer to *Granulicella tundricola* and *Granulicella rosea* (Supplementary Figure S2A). Genome alignment between the strains showed several genomic rearrangements and few regions of genome similarity (Supplementary Figure S2B).

**TABLE 1:** Genomic features of *Granulicella* sp. 5B5 and WH15.

**Genome**	***Granulicella* sp. 5B5**	***Granulicella* sp. WH15**
Size (bp)	3,928,701	4,673,153
G + C content (%)	61.1	60.7
Number of predicted coding sequences	3,306	3,939
Number of features in Subsystems	1,260	1,496
Number of predicted RNA genes	51	51
Number of contigs	1	1

RAST analysis showed that only 37% of the annotated genes (1,260/3,374) could be assigned to subsystems. Among the subsystem categories present in the genome, “carbohydrates” and “dormancy and sporulation” had the highest and lowest feature counts, respectively (Supplementary Figure 2B).

Analysis with ANTISMASH v4.2.0 revealed the presence of five biosynthetic gene clusters (Table 3). The identified clusters showed potential for the production of terpenes, beta-lactone, type III polyketide synthases (T3PKS) and bacteriocin. Only cluster 1 showed similarity to a known cluster (Malleobactin NRPS, 11% similarity). Annotation with dbCAN (Table 2) revealed the presence of 92 carbohydrate-associated enzymes, distributed in four classes: seven carbohydrate esterases (CE), 63 glycosyl hydrolases (GH), 20 glycosyl transferases (GT), and two polysaccharide lyases (PL), but no carbohydrate binding modules (CBM) or auxiliary activities (AA) were observed. Further evaluation of the CAZymes demonstrated the potential for the degradation of a wide range of carbohydrates, as the genome of *Granulicella* sp. 5B5 possessed CDSs for 48 CAZyme families, including as α- and β-glucosidases (EC 3.2.1.20, EC 3.2.1.21) (GH1, GH13, GH3, GH31), α- and β-galactosidases (EC 3.2.1.22, EC 3.2.1.23) (GH2, GH27, GH35, GH57), α- and β-mannosidases (EC 3.2.1.25, EC 3.2.1.25) (GH1, GH38, GH125), α-L-rhamnosidases (EC 3.2.1.40) (GH28, GH106), α-L-fucosidases (EC 3.2.1.51) (GH29), β-xylosidases (EC 3.2.1.37) (GH39, GH43, GH54), α-L-arabinofuranosidases (EC 3.2.1.55) (GH43, GH54) and α-amylases (EC 3.2.1.1) (GH13, GH77). The cellulose synthase genes observed in other *Granulicella* genomes (Rawat et al., 2013a, b) were not observed in the genome of *Granulicella* sp. 5B5.

**TABLE 2:** Number of predicted genes from different CAZyme families observed in the genomes of *Granulicella* sp. 5B5 and WH15.

**CAZyme family**	***Granulicella* sp. 5B5**	***Granulicella* sp. WH15**
Auxiliary activity (AA)	0	13
Carbohydrate binding module (CBM)	0	22
Carbohydrate esterase (CE)	7	41
Cohesin	0	1
Glycoside hydrolase (GH)	63	86
Glycosyl transferase (GT)	20	52
Polysaccharide lyase (PL)	2	2
**Total**	**92**	**217**

**TABLE 3:** Growth of *Granulicella* sp. WH15 and 5B5 in solid culture medium with different metal concentrations.

**Metal source**	**Strain**	**Concentration in mM**					
		**0.5**	**1**	**2**	**5**	**10**	**15–40**
	WH15						
ZnCl_2_		−	−	−	−	−	−
NiCl_2_		+	+	+	−	−	−
MnCl_2_		+	+	+	+	+	+
CoCl_2_		−	−	−	−	−	−
CuCl_2_		−	−	−	−	−	−
NaMoO_4_		+	+	−	−	−	−
AlCl_2_		+	−	−	−	−	−
CdCl_2_		−	−	−	−	−	−
C_8_H_4_K_2_O_12_Sb_2_		−	−	−	−	−	−
	5B5						
ZnCl_2_		+	−	−	−	−	−
NiCl_2_		+	+	+	−	−	−
MnCl_2_		+	+	+	+	+	+
CoCl_2_		−	−	−	−	−	−
CuCl_2_		−	−	−	−	−	−
NaMoO_4_		+	+	−	−	−	−
AlCl_2_		−	−	−	−	−	−
CdCl_2_		−	−	−	−	−	−
C_8_H_4_K_2_O_12_Sb_2_		−	−	−	−	−	−

*+, positive colony formation; −, no growth. Comparisons made with the control without metal.*

### Metal Resistance Assays and Metal Resistance Gene Annotation

Metal resistance tests on agar medium demonstrated that both *Granulicella* strains were able to grow on NiCl_2_ (max 2 mM) and NaMoO_4_ (max 1 mM). *Granulicella* sp. 5B5 was able to grow on 0.5 mM ZnCl_2_, *Granulicella* sp. WH15 grew on 0.5 AlCl_2_, and both strains could grow at concentrations of MnCl_2_ up to 40 mM (Table 3).

*Granulicella* sp. WH15 genome search against BacMet experimentally confirmed and predicted resistance genes databases revealed 28 ORFs and 78 ORFS, respectively, with hits similar (>45% identity) to genes involved in resistance toward a wide range of metals such as As, Cd, Zn, Co, Cu, Fe, Mn, Mo, Ni, and Zn, multidrug and metal efflux transporters and DNA binding response regulators (Supplementary Table S2).

In addition, *Granulicella* sp. WH15 possessed two non-identical copies of Mn transporter *mntH* (TC 2.A.55.3.1) (GWH15_03215 and GWH15_08180), and two ORFs (GWH15_19170 and GWH15_03225), with 60.2 and 44% identity with the Mn transcriptional regulator *mntR*, respectively.

We obtained a similar profile for *Granulicella* sp. 5B5 genome, with 65 ORFs that had hits higher than 45% identity against the experimentally confirmed database and 23 ORFs that had hits higher than 45% identity against the predicted database (Supplementary Table S3). For both searches, predicted genes involved in resistance to several metal ions, as well as multidrug and metal efflux transporters and transcription regulators were observed (Supplementary Table S3). The genome of *Granulicella* sp. 5B5 also contains two non-identical copies of the *mntH* transporter (TC 2.A.55.3.1) (G5B5_04510 and G5B5_07650), and three ORFs (G5B5_10215, G5B5_13625, and G5B5_13770) related to Mn transcriptional regulator *mntR*, as well as three ORFs similar to Mn ABC transporters *mntA*/*ytgA* (TC 3.A.3.3.3) (G5B5_11150 and G5B5_14665) and Mn efflux pump *mntP* (TC 2.A.107.1.1) (G5B5_01505) (Supplementary Table S3).

### Impact of Manganese on Proteome of *Granulicella* Strains

Since Mn had a significant effect on the growth of both *Granulicella* strains, we further investigated the effects of Mn on cellular metabolism by proteomics analysis. At day 4, the differences in growth between control and Mn treatment started to be statistically significant for both strains (Supplementary Figure S3), and therefore samples were collected at this particular time point for proteome analysis.

**FIGURE 3:**
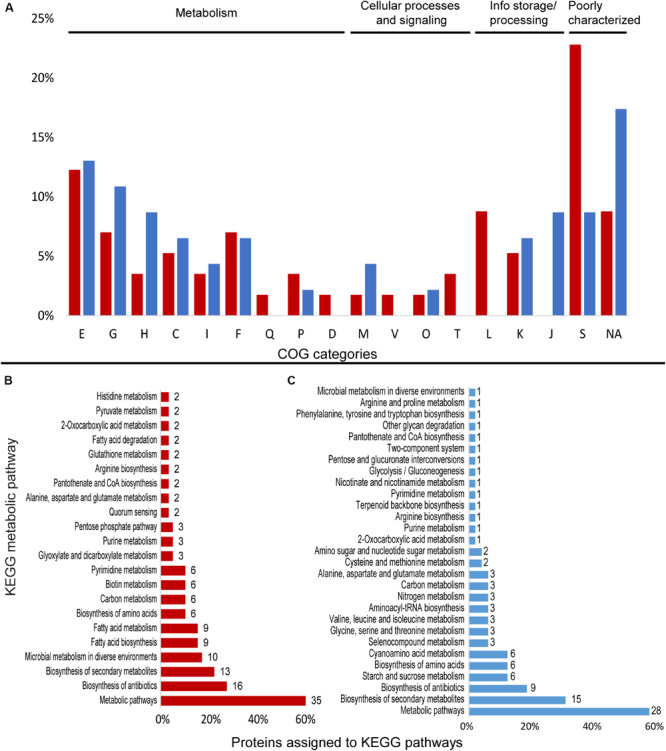
Differentially expressed proteins assigned to COG categories and KEGG pathways in the proteomic profile of *Granulicella* strain 5B5 with the addition of Mn. **(A)** Percentage of upregulated (red) and downregulated (blue) proteins assigned to COG categories; **(B)** number of upregulated proteins assigned to KEGG pathways (only pathways with more than one protein mapped are shown); **(C)** number of downregulated proteins assigned to KEGG pathways. E-amino acid transport and metabolism; G-carbohydrate transport and metabolism; H-coenzyme transport and metabolism; C-energy production and conversion; I-lipid transport and metabolism; F-nucleotide transport and metabolism; Q-secondary metabolites; D-cell cycle; N-cell motility; M-cell wall/membrane/envelope biogenesis; V-defense mechanisms; P-inorganic ion transport and metabolism; U-intracellular trafficking; O-post translational modification; T-signal transduction mechanisms; L-replication, recombination and repair; K-transcription; J-translation; S-function unknown; R-general function and prediction; X-mobilome; NA-not assigned.

Proteome data for *Granulicella* sp. 5B5 showed that 1,028 proteins were detected in both treatments in at least two out of three replicates each. Overall, 216 proteins showed significantly different abundances, with 14 so-called on/off proteins, which were present in only one condition (Supplementary Figure S4A). The proteome patterns of *Granulicella* sp. 5B5 under control and Mn treatments are depicted in Supplementary Figure S5A. A total of 46 proteins were upregulated 1.5-fold and 11 proteins were “on,” while 43 proteins were downregulated 1.5-fold and 3 proteins were “off” in the Mn treatment. Among these differentially expressed proteins, 90 could be assigned to COG categories and 67 could be annotated to KEGG orthologs (Figure 3 and Supplementary Table S4).

The qualitative analysis of the proteomic data for *Granulicella* sp. WH15 demonstrated that, overall, 909 proteins were identified in both conditions in two out of three replicates each. In total, 171 proteins showed significant differences between Mn and control conditions (*t*-test, *p* = 0.01) (Supplementary Figure S4B). The proteome patterns of *Granulicella* sp. WH15 under control and Mn treatments are depicted in Supplementary Figure S5B.

**FIGURE 4:**
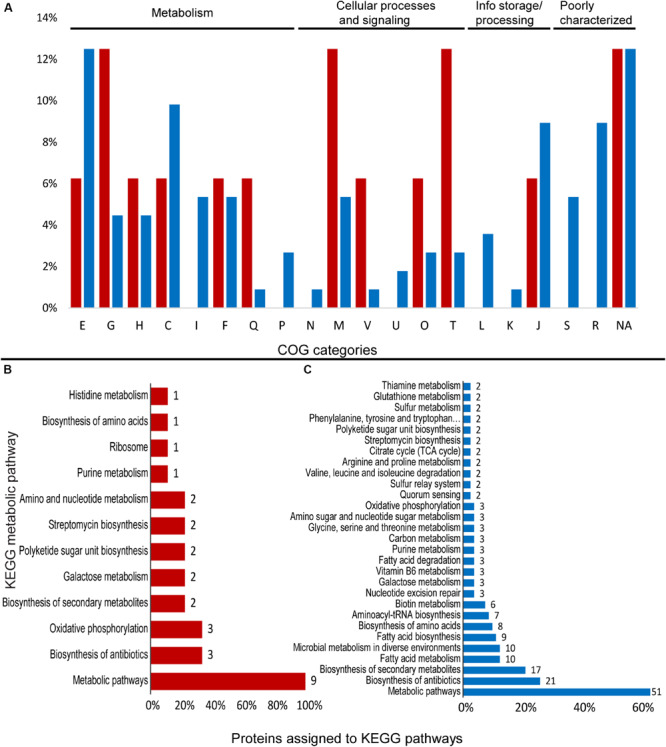
Differentially expressed proteins assigned to COG categories and KEGG pathways in the proteomic profile of *Granulicella* strain WH15 with the addition of Mn. **(A)** Percentage of upregulated (red) and downregulated (blue) proteins assigned to COG categories; **(B)** number of upregulated proteins assigned to KEGG pathways; **(C)** number of downregulated proteins assigned to KEGG pathways (only pathways with more than one protein mapped are shown). E-amino acid transport and metabolism; G-carbohydrate transport and metabolism; H-coenzyme transport and metabolism; C-energy production and conversion; I-lipid transport and metabolism; F-nucleotide transport and metabolism; Q-secondary metabolites; D-cell cycle; N-cell motility; M-cell wall/membrane/envelope biogenesis; V-defense mechanisms; P-inorganic ion transport and metabolism; U-intracellular trafficking; O-post translational modification; T-signal transduction mechanisms; L-replication, recombination and repair; K-transcription; J-translation; S-function unknown; R-general function and prediction; X-mobilome.; NA-not assigned.

Comparisons between treatments showed that 16 proteins were upregulated at least 1.5-fold, while 93 proteins were downregulated at least 1.5-fold. In addition, 19 proteins were “off” in the Mn treatment and present only in control conditions. Among the significantly different proteins, 112 were assigned to COG categories and 89 were annotated to KEGG orthologs (Figure 4 and Supplementary Table S4).

**FIGURE 5:**
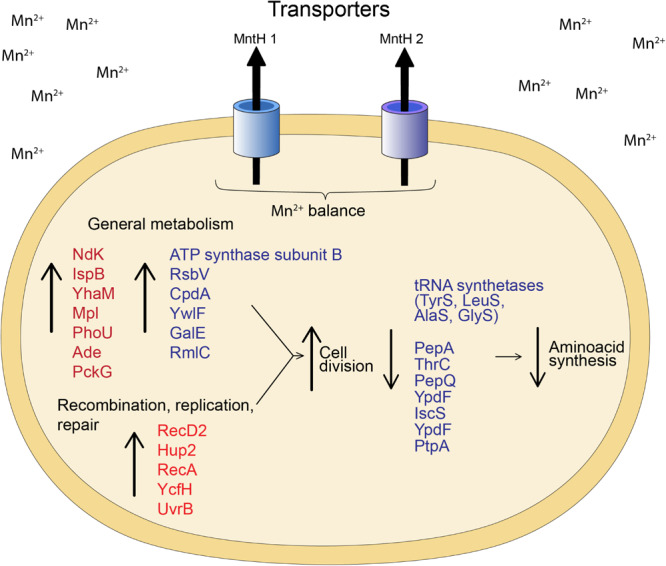
Model of the cellular processes involved in Mn adaptation in *Granulicella* sp. WH15 and *Granulicella* sp. 5B5. Proteins in red were upregulated in *Granulicella* sp. 5B5, proteins in blue were up or downregulated in *Granulicella* sp. 5B5. The arrows depict upregulation (↑) and downregulation (↓) of proteins.

Comparatively, proteome analysis revealed different responses to Mn for the two strains. *Granulicella* sp. 5B5 had more upregulated proteins (57), while *Granulicella* sp. WH15 had more downregulated proteins (112). Further comparisons demonstrated that no upregulated or downregulated proteins were shared between strains. In *Granulicella* sp. 5B5, a higher number of upregulated proteins that can use Mn^2+^ as co-factor was detected. For both strains, proteins annotated as Mn transporters were not detected, probably because the method used for protein extraction was not specific for membrane proteins. A model summarizing the cellular processes involved in Mn adaptation for both *Granulicella* sp. WH15 and *Granulicella* sp. 5B5 is depicted in Figure 5.

### Upregulated Proteins in Response to Manganese

#### *Granulicella* sp. 5B5

Cluster of Ortholog Groups analysis showed that proteins were mainly distributed among the categories E-amino acid transport and metabolism (7), L-replication, recombination and repair (5), G-carbohydrate transport and metabolism (4), and F-nucleotide transport and metabolism (4) (Figure 3A). A total of 36 proteins were assigned to KEGG orthologs, that were mapped to 47 metabolic pathways, and some orthologs were mapped to more than one pathway. The majority of the proteins were mapped to “general” metabolic pathways (35), biosynthesis of antibiotics (16), biosynthesis of secondary metabolites (13), and microbial metabolism in diverse environments (10) (Figure 3B), but no metabolic pathway was specifically upregulated. Looking deeper into the upregulated proteins, we identified several enzymes that require Mn^2+^ or Mg^2+^ as cofactor, such as nucleoside diphosphate kinase Ndk (EC 2.7.4.6), octaprenyl diphosphate synthase IspB (EC 2.5.1.90), UDP-N-acetylmuramate–L-alanyl-gamma-D-glutamyl-meso-2,6-diaminoheptandioate ligase Mpl (EC 6.3.2.45), adenine deaminase Ade (EC 3.5.4.2), phosphate-specific transport system accessory protein PhoU, oxalate decarboxylase OxdD (EC 4.1.1.2), phosphoenolpyruvate carboxykinase pckG (EC 4.1.1.32) and 3′-5′ exoribonuclease YhaM (EC:3.1.-.-) (Supplementary Table S4).

Search against BacMet Databases showed 25 proteins with hits (>45% identity) against the experimentally confirmed database and 17 proteins with hits (>45% identity) against the predicted metal resistance genes database (Supplementary Table S5). The genes were mostly associated resistance/homeostasis of several metal ions, such as Fe, Cu, As, Ni, Co, and Zn. Interestingly, four ORFS were similar to metal ion transporters that could be involved in Mn homeostasis: ORF_05650 (hypothetical protein) had 43.5% identity with copper-translocating P-type ATPase CueA (TC 3.A.3.5.39); ORF_03225 (hypothetical protein) had 31% identity with copper-translocating P-type ATPase CopA (TC 3.A.3.5.1); ORF_06495 (TcrA) had 40% identity with copper-translocating P-type ATPase CopA (TC 3.A.3.5.1) and ORF_14875 (NatA_2) had 31% identity with metal ABC transporter ATP-binding protein TroB (TC 3.A.1.15.8) (Supplementary Table S5).

#### *Granulicella* sp. WH15

Cluster of Ortholog Groups analysis showed that upregulated proteins were distributed within several COG categories. The most common categories were: “G-carbohydrate transport and metabolism” (2), “M-cell wall/envelope/membrane biogenesis” (2), and “T-signal transduction mechanisms” (2) (Figure 4A). Within KEGG metabolic pathways, no complete pathway upregulation was observed.

A total of 9 upregulated proteins were assigned to KEGG orthologs, which were mapped to 12 KEGG metabolic pathways, since some orthologs were mapped to more than one pathway. Most of the annotated proteins were mapped to “general” metabolic pathways (9), biosynthesis of antibiotics (3), and oxidative phosphorylation (3) (Figure 4B).

Some of the upregulated proteins were ATP synthase subunit b, and the carbohydrate-associated enzymes putative sugar phosphate isomerase YwlF (EC 5.3.1.-), UDP-glucose 4-epimerase GalE (EC 5.1.3.2) and dTP-4-dehydrorhamnose 3,5 epimerase RmlC (EC 5.1.3.13) (Supplementary Table S4). The search against BacMet databases showed that three ORFs had hits against genes related to metal ion homeostasis. ORF GWH15_13825 (*cysO*) had 42% identity with predicted resistance gene *copA*, encoding a copper-exporting P-type ATPase (TC 3.A.3.5.1) and 31% similarity with the experimentally confirmed cation/multidrug efflux pump AdeG (TC 2.A.6.2.44), which is part of AdeFGH efflux system. ORF GWH15_17845 (hypothetical protein) had 30% similarity with predicted resistance gene *rcnB/yohN* (TC 2.A.113.1.1), a nickel/cobalt homeostasis protein; ORF GWH15_19690 (hypothetical protein) has 36% identity with predicted resistance gene *mtrA*, a DNA-binding response regulator.

### Downregulated Proteins in Response to Manganese

#### *Granulicella* sp. 5B5

Annotation with COG database demonstrated that most proteins were distributed within the categories E-amino acid transport and metabolism (6), G-carbohydrate transport and metabolism (5), H-coenzyme transport and metabolism (4), and J-translation, ribosomal structure and biogenesis (4) (Figure 3A). Overall, 31 proteins were assigned to KEGG identifiers, which were mapped to 29 KEGG metabolic pathways. Most of the proteins were mapped to “general” metabolic pathways (29), biosynthesis of secondary metabolites (15), and biosynthesis of antibiotics (9) (Figure 3B). Several proteins linked to general metabolism were repressed, but no specific metabolic pathway seemed to be repressed. Among the repressed proteins we observed enzymes involved in various cellular functions, such as cysteine synthase CysM (EC 2.5.1.47), L-threonine dehydratase TdcB (EC 4.3.1.19), ribonucleoside-diphosphate reductase subunit beta NrdB (EC 1.17.4.1), putative glucose-6-phosphate 1-epimerase YeaD (EC 5.1.3.15) and carbonic anhydrase CynT (EC 4.2.1.1) (Supplementary Table S4).

#### *Granulicella* sp. WH15

Within COG categories, most of the downregulated proteins belonged to the categories “E-aminoacid transport and metabolism” (14), “C-energy production and conversion” (11), “J-Translation, ribosomal structure and biogenesis” (10), and “R-General function prediction” (10) (Figure 4A). Overall, 80 proteins were assigned to KEGG orthologs, which were mapped to 52 KEGG metabolic pathways, and some orthologs were mapped to more than one type of pathway. The majority of the proteins were mapped to “general” metabolic pathways (51), biosynthesis of antibiotics (21), biosynthesis of secondary metabolites (17), fatty acid metabolism (10), and microbial metabolism in diverse environments (10) (Figure 4B), with no pathway specifically stimulated. Several enzymes involved in amino acid biosynthesis and metabolism were identified, such as tyrosyl, leucyl, alanyl, and glycyl-tRNA synthetases (EC 6.1.1.-) (TyrS, LeuS, AlaS, and GlyS), leucyl aminopeptidase PepA (EC 3.4.11.1), threonine synthase ThrC (EC 4.2.3.1), xaa-pro-dipeptidyl-aminopeptidase PepQ (EC 3.4.13.9), aminopeptidase YpdF (EC 3.4.11.-), cysteine desulfurase IscS (EC 2.8.1.7), and prolyl tripeptidyl peptidase PtpA (EC 3.1.3.48) (Supplementary Table S4).

## Discussion

In this study, we evaluated the effect of trace element addition on the growth of two strains of *Granulicella*, belonging to phylum *Acidobacteria* class “Acidobacteriia.” We observed that the growth in liquid medium of both strains was improved by the addition of Mn, to which the strains tolerated relatively higher concentrations in comparison to other metal salts. Furthermore, variations in tolerance to metal salt concentrations suggest that the *Granulicella* spp. 5B5 and WH15 possess different mechanisms to deal with metal ion homeostasis and stress, also reflecting the low similarity observed in the genome alignment between both strains. *Granulicella* sp. 5B5 is likely more adapted to survive in an environment with higher concentration of several metal ions when compared to *Granulicella* sp. WH15.

When evaluated separately, Mn salt had a more pronounced effect than other metal salts, but the mix of metals was more effective in enhancing bacterial growth, reflecting wide physiological needs and the importance of different metal ions in bacterial metabolism. For instance, *E. coli* BW25113 growth was maximized with a mixture of Ni and Fe, which had a better effect than each metal separately and other metal salt mixtures (Trchounian et al., 2016). The amendment of Mn to fermentation medium improved the growth of *Lactobacillus bifermentans*, increasing the production and activity of the enzyme glucose isomerase, necessary for biotechnological applications (Givry and Duchiron, 2008). Manganese is also an essential growth factor for *Lactobacillus casei* and other species of lactobacilli, which is attributed to its role as a co-factor of enzyme lactate dehydrogenase, enhancing cell growth rate and biomass concentration (Fitzpatrick et al., 2001; Lew et al., 2013). On the other hand, Mn had no significant impact in the growth of *Halobacterium* (Joshi et al., 2015).

Among the metals used for metal salt tolerance testing, *Granulicella* spp. WH15 and 5B5 only showed tolerance to Mn, exhibiting growth at the concentration of 40 mM Mn, which is higher than typically observed for most bacterial strains. For instance, a resistant *Serratia marcescens* strain, isolated from Mn mine waters in Brazil, could grow on a maximum concentration of 6 mM Mn (Barboza et al., 2017). Mn tolerance can vary widely in microorganisms, with a minimal inhibitory concentration ranging from 0.1 to 228.9 mM Mn in certain marine bacterial strains (Gillard et al., 2019). Comparisons with other strains, however, should be interpreted cautiously, since culture media composition differ among studies, and it was already observed that organic compounds in nutrient agar can chelate metals, decreasing their availability for the microorganisms tested (Polti et al., 2007; El Baz et al., 2015).

Manganese is essential for the growth and survival of living organisms. It is a co-factor of a wide range of enzymes, being vital in specific metabolic pathways, such as sugar, lipid, and protein catabolism (Jensen and Jensen, 2014), oxygenic photosynthesis in cyanobacteria (Kehres and Maguire, 2003; Cvetkovic et al., 2010), signal transduction, stringent response, sporulation, and pathogenesis (Kehres and Maguire, 2003; Jensen and Jensen, 2014). One of the most widely known and studied Mn functions is the detoxification of ROS, where it is a redox-active co-factor in free radical detoxifying enzymes, such as Mn-superoxide dismutase (MnSod) and mangani-catalase (Jakubovics and Jenkinson, 2001; Jensen and Jensen, 2014). Additionally, the detoxifying capaticities of Mn are not only enzyme-mediated, since non-protein complexes of Mn can also work as antioxidants when enzymes are not sufficient (Jensen and Jensen, 2014). Nonetheless, Mn homeostasis needs a refined balance, since Mn can bind to Mg-binding sites in enzymes and regulators, causing mismetallation, which can have deleterious effects in the cells (Hohle and O’Brian, 2014; Chandrangsu et al., 2017). Both *Granulicella* strains were isolated from decaying wood material, in association with the white rot fungus *H. fasciculare* (Valášková et al., 2009), where topsoil-litter samples have Mn concentrations as high as 101 mg Mn/kg (Costa et al., 2020a). Since high concentrations of Mn can be observed in wood decay environments, especially when decomposition is caused by white rot fungi (Blanchette, 1984), tolerance to higher manganese concentrations could be a strategy to assure the survival of the studied *Acidobacteria* in this environment.

The evaluation of the predicted genes in both did not reveal the presence of common genes involved in Mn regulation, such as the transcriptional regulator mntR (Jensen and Jensen, 2014). This result implies that the homeostasis of Mn in *Granulicella* strains is under control of another transcriptional regulator. MntR is a Mn-dependent DtxR family member that controls the expression of Mn uptake systems *mntABCD* and *mntH* in *Bacillus subtilis* and *Salmonella enterica* (Que and Helmann, 2002; Kehres and Maguire, 2003), for instance. However, it appears that MntR is absent in *Rhizobiales* and *Rhodobacterales*, in which the function is replaced by the protein Mur (van Bakel and Wijmenga, 2005). In addition, MntR-independent regulation of Mn homeostasis has been proposed (Modi et al., 2011; Kaur et al., 2014). Nonetheless, both strains possessed two non-identical copies of the Mn transporter *mntH*. MntH seems to be the main responsible transporter in Mn influx, but it was already observed that Mn has a significant repressive effect in the expression of Mn transporters under manganese sufficiency, keeping Mn homeostasis and optimal levels of Mn inside the cells (Guedon et al., 2003; Jensen and Jensen, 2014). Furthermore, the search of the genomes against BacMet databases demonstrated that both genomes possessed a wide range of transporters that are linked to the homeostasis of diverse metal ions.

Within the upregulated proteins from *Granulicella* sp. 5B5, three proteins were similar to copper P-type ATPase transporters and one protein was similar to the metal ion ATP-binding ABC transporter TroB. P-type ATPases, such as CtpC, in *Mycobacterium* species are responsible not only for Mn efflux, removing excess metal ion from the cells, but are also required for the metalation of proteins (Padilla-Benavides et al., 2013). ABC transporters are as well responsible for Mn uptake, important in several bacterial species (Papp-Wallace and Maguire, 2006; Jensen and Jensen, 2014). In the proteome profile of *Granulicella* sp. WH15, we similarly observed a protein similar to the copper-exporting P-type ATPase and cation/multidrug efflux pump AdeG, which could be involved in maintaining optimal levels of Mn inside the bacterial cell. Furthermore, we found a protein similar to RcnB/YohN, which is an essential protein for Ni/Co homeostasis in *E. coli* (Bleriot et al., 2011), and a protein similar to gene *MtrA*, which is involved in cell division control and cell wall metabolism in *Mycobacterium tuberculosis* (Gorla et al., 2018).

In addition to transporters, the proteomic analyses of both bacteria strains revealed other proteins which might be involved in the growth enhancement of both strains. The proteomic profile of *Granulicella* sp. WH15 had fewer upregulated enzymes in comparison to *Granulicella* sp. 5B5, demonstrating that Mn had more impact in transcription and protein expression in *Granulicella* sp. 5B5. Several enzymes that require Mn or Mg as cofactor were upregulated in the proteome of *Granulicella* sp. 5B5, but no enhancement of specific metabolic pathway was detected. Overall, the upregulated enzymes in *Granulicella* sp. 5B5 were involved in general metabolic pathways, which could be stimulated due to the higher metabolism necessary for a faster growth. For instance, nucleoside diphosphate kinase Ndk is a critical enzyme involved in the nucleotide metabolism of microorganisms, but is also part of posttranslational modification of proteins, as well as regulation of genes linked to quorum sensing, proteases and toxins (Yu et al., 2017). Octaprenyl diphosphate synthase IspB is involved in the production of the lateral chain of ubiquinones, and is an essential enzyme for respiration and normal growth of *E. coli* (Okada et al., 1997). 3′-5′ exoribonuclease YhaM was identified as a participating enzyme in mRNA turnover in *B. subtilis* (Oussenko et al., 2005). UDP-N-acetylmuramate–L-alanyl-gamma-D-glutamyl-meso-2,6-diaminoheptandioate ligase Mpl is a recycling enzyme that allows the constant remodeling of bacterial cell wall polymer occurring during cell growth and division (Herve et al., 2007). Moreover, the category replication, recombination and repair were only present within upregulated proteins, with proteins DNA helicase RecD2, DNA -binding protein Hup 2, protein RecA, metal-dependent hydrolase YcfH and UvrABC system protein UvrB. In addition, no specific pathway seemed to be repressed.

In the proteomic profile of *Granulicella* sp. WH15, we observed the upregulation enzymes involved in energy production, amino acid metabolism and transcription regulation. For instance, ATP synthase subunit β is part of the ATP synthase complex, which is involved in ATP synthesis and hydrolysis (Sokolov et al., 1999). ATP phosphoribosyltransferase is an enzyme involved in histidine biosynthesis, a reaction that requires Mg, which can be substituted by Mn (Tebar et al., 1977). Protein RsbV is a positive regulator of factor sigma β, which, in Gram-positive bacteria is a key contributor to the resilience and survival of bacterial species to environmental conditions, such as variations in pH, osmotic stress or entry into stationary growth phase (Kullik and Giachino, 1997; Guldimann et al., 2016). Phosphodiesterase CpdA is responsible for the hydrolysis of the second messenger cyclic AMP (cAMP), which controls cell responses to a variety of environmental conditions (Fuchs et al., 2010). Similarly to the response of *S. marcescens* (Queiroz et al., 2018) to Mn stimulation, the limited number of upregulated proteins from *Granulicella* sp. WH15 could be attributed to the adaptation to the concentration of Mn used in the experiment, since it is an optimal growth condition. Furthermore, as a co-factor of several important enzymes (Crowley et al., 2000; Jensen and Jensen, 2014), the presence of manganese might be improving bacterial growth by activating enzymes and enhancing metabolic activities involved in cell cycle and division, even when the enzymes were not differentially expressed. Among the repressed proteins, we found several amino acid synthases, that could be inhibited due to the ready availability of amino acids in the culture medium composition (Mader et al., 2002), supplied by yeast extract.

The proteomic profiles of both strains did not exhibit the overexpression of specific pathways, indicating that Mn was more important in enhance enzymatic activity than to protein expression regulation. Finally, we did not find the most common transcriptional regulation of Mn homeostasis, implying that Mn regulation is performed by a different gene or set of genes, but our analysis revealed candidate transporters that could be potentially involved in Mn homeostasis for *Granulicella* species. The presence of such type of transporters could facilitate the uptake of metal ions, improving the adaptability of bacteria to metal enriched environments (Campanharo et al., 2016), promoting a tight regulation of metal ion homeostasis, as well as a tolerance to higher concentrations of metals.

## Data Availability Statement

The *Granulicella* sp. 5B5 strain genome is deposited at NCBI with accession number CP046444. The *Granulicella* sp. WH15 strain genome is deposited at NCBI with accession number CP042596. The mass spectrometry proteomics data were deposited to the ProteomeXchange Consortium (http://proteomecentral.proteomexchange.org) via the PRIDE (Perez-Riverol et al., 2019) partner repository with the dataset identifier PXD016551.

## Author Contributions

EK and OC: conceptualization. OC, CO, DZ, CW, and KR: methodology. OC, EK, and DZ: data analysis and integration. OC: writing – original draft preparation. EK, OC, CB, and DZ: writing – review and editing. All authors have read and agreed to the published version of the manuscript.

## Conflict of Interest

The authors declare that the research was conducted in the absence of any commercial or financial relationships that could be construed as a potential conflict of interest.

## Footnotes

1 http://rast.nmpdr.org/

2 http://bcb.unl.edu/dbCAN2/

3 http://eggnogdb.embl.de/#/app/emapper
