# Supplementary material for: Impact of Different Trace Elements on the Growth and Proteome of Two Strains of Granulicella, Class “Acidobacteriia”
Source: Front Microbiol. 2020 Jun 18;11:1227. doi: 10.3389/fmicb.2020.01227 (PMC7315648; doi:10.3389/fmicb.2020.01227)
Supplement: Supplementary file 1 [file Data_Sheet_1.docx]

Supplementary Material

# Supplementary Tables S1, S2, S3, S5.

Table S1: Composition of trace element solution SL10 and final concentration (µM) of each individual metal in culture medium

| SL10 composition | Final µM concentration in culture medium |
| --- | --- |
| FeCl_2_.4H_2_O | 7.54 |
| ZnCl_2_ | 0.51 |
| MnCl_2_.4H_2_O | 0.51 |
| H_3_BO_3_ | 0.10 |
| CoCl_2_.6H_2_O | 0.80 |
| CuCl_2_.2H_2_O | 0.01 |
| NiCl_2_.6H_2_O | 0.10 |
| Na_2_MoO_4_.2H_2_O | 0.15 |

| Table S2: *Granulicella* strain WH15 gene annotation with BacMet databases. Annotation performed against the experimentally confirmed and predicted resistance genes databases using BacMet Scan software. Only genes with hits ≥ 40% identity in one of the databases are shown. | | | | | | |
| --- | --- | --- | --- | --- | --- | --- |
| ORF | EXP | Compound | Iden. | PRE | Compound | Identity |
| GWH15_16385 | *arsB* | As | 63.5 | *acr3* | Arsenical-resistance protein | 70.3 |
| GWH15_11300 | *mdtB* | Zn | 60.5 | *mdtB/yegN* | Multidrug transporter subunit MdtB | 60.6 |
| GWH15_16465 | *hoxN* | Ni | 60.0 | *hoxN* | HoxN/HupN/NixA family Ni/Co transporter | 60.0 |
| GWH15_00760 | *acn* | Fe | 59.5 | *acn* | Aconitate hydratase | 60.1 |
| GWH15_02935 | *mdtB/yegN* | SDC, HCL | 58.5 | *mdtB/yegN* | Multidrug transporter subunit MdtB | 58.6 |
| GWH15_12080 | *mdtB/yegN* | SDC, HCL | 58.5 | *mdtB* | Multidrug transporter subunit MdtB | 59.2 |
| GWH15_00470 | *ruvB* | Cr, Te, Se | 58.5 | *ruvB* | DNA helicase RuvB | 59.6 |
| GWH15_11305 | *mdtC/yegO* | SDC and other | 56.5 | *mdtC/yegO* | Multidrug transporter subunit MdtC | 56.6 |
| GWH15_13565 | *pstB* | As | 56.2 | *pstB* | Phosphate ABC transporter ATP-binding protein PstB | 56.6 |
| GWH15_16395 | *arsM* | As | 54.4 | *arsM* | Arsenite S-adenosylmethyltransferase | 43.8 |
| GWH15_02930 | *mdtC/yegO* | SDC and other | 52.9 | *mdtC/yegO* | Multidrug transporter subunit MdtC | 52.8 |
| GWH15_12085 | *mdtC/yegO* | SDC and other | 52.5 | *mdtC* | Multidrug transporter subunit MdtC | 52.7 |
| GWH15_18655 | *sodA* | H2O2 | 51.3 | *sodA* | Superoxide dismutase | 51.3 |
| GWH15_12800 | *mdtC/yegO* | SDC and other | 51.3 | *mdtC* | Multidrug transporter subunit MdtC | 51.2 |
| GWH15_02405 | *galE* | CTAB | 50.9 | *galE* | UDP-glucose 4-epimerase GalE | 55.9 |
| GWH15_16550 | *pcoA* | Cu | 49.8 | *copA* | Cu resistance system multicopper oxidase | 51.8 |
| GWH15_04740 | *arsT* | As | 49.8 | *arsT* | Thioredoxin-disulfide reductase | 53.0 |
| GWH15_06695 | *acrR/ybaH* | Acriflavine | 48.3 | *-* | - | - |
| GWH15_05790 | *cusA/ybdE* | Cu, Ag | 48.3 | *cusA/ybdE* | CusA/CzcA family heavy metal efflux RND transporter | 48.3 |
| GWH15_15485 | *fabI* | Triclosan | 48.2 | *fabI* | Enoyl-ACP reductase FabI | 50.2 |
| GWH15_10040 | *zraR/hydH* | Zn | 47.7 | *zraR/hydH* | Two-component system response regulator ZraR | 49.2 |
| GWH15_04095 | *cpxR* | H2O2 and other | 46.5 | *cpxR* | DNA-binding response regulator | 50.0 |
| GWH15_04345 | *dmeR* | Co, Ni | 46.2 | *dmeR* | Metal/formaldehyde-sensitive transcriptional repressor | 46.2 |
| GWH15_19170 | - | - | - | *mntR* | Inner membrane protein YbiR | 60.2 |
| GWH15_14955 | - | - | - | *mtrD* | Phospho-N-acetylmuramoyl-pentapeptide-transferase | 58.4 |
| GWH15_15295 | - | - | - | *glpF* | Glycerol kinase | 56.8 |
| GWH15_05235 | - | - | - | *merA* | Mercury(II) reductase | 56.3 |
| GWH15_15725 | - | - | - | *ruvB* | DNA helicase RuvB | 54.8 |
| GWH15_14350 | - | - | - | *aioE* | NAD(P)/FAD-dependent oxidoreductase | 54.3 |
| GWH15_16410 | - | - | - | *arsC* | Arsenate reductase ArsC | 54.0 |
| GWH15_03545 | - | - | - | *recG* | ATP-dependent DNA helicase RecG | 52.8 |
| GWH15_17515 | - | - | - | *merA* | Mercury(II) reductase | 52.6 |
| GWH15_00860 | - | - | - | *aioE* | NAD(P)/FAD-dependent oxidoreductase | 52.3 |
| GWH15_11710 | - | - | - | *trgB* | Te resistance protein | 51.6 |
| GWH15_09530 | - | - | - | *zupT/ygiE* | Zn transporter ZupT | 51.1 |
| GWH15_01540 | - | - | - | *actA* | Apolipoprotein N-acyltransferase | 50.0 |
| GWH15_10285 | - | - | - | *copR* | DNA-binding response regulator | 50.0 |
| GWH15_12250 | - | - | - | *arsT* | Thioredoxin reductase | 49.5 |
| GWH15_16485 | - | - | - | *emrBsm* | MFS transporter | 48.8 |
| GWH15_08215 | - | - | - | *trgB* | Te resistance protein | 48.6 |
| GWH15_19280 | - | - | - | *copG* | Uncharacterized conserved protein | 48.6 |
| GWH15_07930 | - | - | - | *copC* | Cu resistance protein | 48.6 |
| GWH15_03770 | - | - | - | *emhA* | Efflux RND transporter periplasmic adaptor subunit | 48.6 |
| GWH15_04310 | - | - | - | *zupT/ygiE* | Zn transporter ZupT | 48.6 |
| GWH15_17565 | - | - | - | *arsT* | thioredoxin-disulfide reductase | 48.1 |
| GWH15_00030 | - | - | - | *modC* | ABC transporter ATP-binding protein | 47.7 |
| GWH15_07970 | - | - | - | cop-unnamed | Cu oxidase | 47.7 |
| GWH15_10290 | *copR* | Cu | 44.4 | *copR* | DNA-binding response regulator | 47.6 |
| GWH15_12715 | - | - | - | *mdeA* | Methionine gamma-lyase | 47.6 |
| GWH15_16960 | - | - | - | *fbpC* | ABC transporter ATP-binding protein | 47.6 |
| GWH15_07190 | - | - | - | *gadC/xa*sA | Glutamate:gamma-aminobutyrate antiporter | 47.5 |
| GWH15_00895 | - | - | - | *arsT* | Thioredoxin-disulfide reductase | 47.4 |
| GWH15_08115 | - | - | - | *chrA* | Chromate transporter | 47.4 |
| GWH15_13100 | - | - | - | *vexD* | AcrB/AcrD/AcrF family protein | 47.4 |
| GWH15_16860 | - | - | - | *copB* | Cu transporting ATPase | 47.2 |
| GWH15_18070 | - | - | - | *copS* | Two-component sensor histidine kinase | 47.2 |
| GWH15_02325 | - | - | - | *vmeG* | Efflux RND transporter periplasmic adaptor subunit | 47.1 |
| GWH15_19630 | - | - | - | *arsA* | As pump-driving ATPase | 47.1 |
| GWH15_10660 | *corR* | Cu | 43.8 | *corR* | Sigma-54-dependent Fis family transcriptional regulator | 46.9 |
| GWH15_11005 | - | - | - | *abeS* | Multidrug transporter | 46.9 |
| GWH15_12005 | - | - | - | *soxR* | Redox-sensitive transcriptional activator SoxR | 46.9 |
| GWH15_17680 | - | - | - | *arsM* | Polymerase 2 ADP-ribosyltransferase 2 | 46.8 |
| GWH15_11640 | - | - | - | *abeS* | Multidrug transporter | 46.7 |
| GWH15_03945 | *copR* | Cu | 44.9 | *copR* | DNA-binding response regulator | 46.6 |
| GWH15_05310 | - | - | - | *nhlF* | NHLF_RHORH RecName: Co transport protein NhlF | 46.5 |
| GWH15_02985 | - | - | - | *golT* | Cu-translocating P-type ATPase | 46.3 |
| GWH15_08255 | - | - | - | *pgpA/ltpgpA* | Putative ATP-binding cassette protein subfamily C, member 1 | 45.9 |
| GWH15_01935 | - | - | - | *ctpV* | Metal cation transporter P-type ATPase CtpV | 45.8 |
| GWH15_03140 | - | - | - | *furA* | Transcriptional repressor | 45.7 |
| GWH15_17160 | *comR/ycfQ* | Cu | 43.9 | *comR/ycfQ* | TetR/AcrR family transcriptional regulator | 45.6 |
| GWH15_02610 | - | - | - | *copB* | Cu-exporting ATPase | 45.5 |
| GWH15_13575 | *pstC* | As | 42.2 | *pstC* | Phosphate transporter permease subunit PstC | 45.3 |
| GWH15_13260 | - | - | - | *copS* | Two-component sensor histidine kinase | 45.3 |
| GWH15_14490 | - | - | - | *perR* | Transcriptional repressor | 45.2 |
| GWH15_00410 | - | - | - | *copA* | Putative cadmium-transporting P-type ATPase | 45.2 |
| GWH15_04685 | - | - | - | *fabL/ygaA* | Enoyl-acyl-carrier-protein | 45.2 |
| GWH15_07310 | - | - | - | *cutO* | Cu oxidase | 45.2 |
| GWH15_09900 | - | - | - | *merA* | Mercury(II) reductase | 45.2 |
| GWH15_00360 | - | - | - | *mntR* | Inner membrane protein YbiR | 45.0 |

| Table S3: *Granulicella* strain 5B5 gene annotation with BacMet databases. Annotation performed against the experimentally confirmed and predicted resistance genes databases using BacMet Scan software. Only genes with hits ≥ 40% identity in one of the databases are shown. | | | | | | |
| --- | --- | --- | --- | --- | --- | --- |
| ORF | EXP | Compound | Iden | PRE | Compound | Identity |
| G5B5_03005 | *arsB* | As, Sb | 63.7 | *acr3* | arsenical-resistance protein | 70.5 |
| G5B5_06155 | *hoxN* | Ni | 60.3 | *hoxN* | HoxN/HupN/NixA family Ni/Co transporter | 60.3 |
| G5B5_10055 | *ruvB* | Cr, Te, Se | 59.6 | *ruvB* | DNA helicase RuvB | 59.0 |
| G5B5_07635 | *acn* | Fe | 58.6 | *acn* | Aconitate hydratase | 59.3 |
| G5B5_15620 | *silP* | Ag | 58.2 | *actP* | Cu-transporting P-type ATPase | 66.0 |
| G5B5_12540 | *pstB* | As | 55.8 | *pstB* | ABC transporter ATP-binding protein PstB | 56.6 |
| G5B5_06515 | *mdtC/yegO* | SDC | 55.7 | *mdtC/yegO* | Multidrug transporter subunit MdtC | 55.8 |
| G5B5_06520 | *mdtB/yegN* | SDC | 55.0 | *mdtB* | Multidrug transporter subunit MdtB | 55.4 |
| G5B5_16735 | *merA* | Hg | 52.8 | *merA* | Mercury(II) reductase | 36.4 |
| G5B5_10440 | *aioE* | As | 51.3 | *aioE* | NAD(P)/FAD-dependent oxidoreductase | 51.3 |
| G5B5_07160 | *sodA* | Se, H2O2 | 51.2 | *sodA* | Superoxide dismutase | 54.0 |
| G5B5_13030 | *galE* | CTAB | 50.5 | *galE* | UDP-glucose 4-epimerase GalE | 52.3 |
| G5B5_01815 | *dmeR* | Co, Ni | 50.0 | *dmeR* | Metal sensitive transcriptional repressor | 52.0 |
| G5B5_04025 | *smdA* | DAPI | 50.0 | - | - | - |
| G5B5_06995 | *copR* | Cu | 50.0 | *copR* | DNA-binding response regulator | 52.7 |
| G5B5_07595 | *mtrD* | Triton X-100 | 50.0 | *chrC* | hypothetical protein AYO46_00620 | 32.9 |
| G5B5_08045 | *arsB* | As, Sb | 50.0 | - | - | - |
| G5B5_11195 | *pitA* | Zn, Te | 50.0 | - | - | - |
| G5B5_14705 | *copR* | Co | 49.3 | *copR* | DNA-binding response regulator | 51.1 |
| G5B5_14745 | *frnE* | Cd, H2O2 | 48.7 | - | - | - |
| G5B5_01735 | *pbrD* | Pb | 48.6 | - | - | - |
| G5B5_01970 | *dmeF* | Co, Cd, Ni | 48.5 | - | - | - |
| G5B5_13805 | *mntA/ytgA* | Mn, Cd | 48.5 | - | - | - |
| G5B5_04635 | *iclR* | Sodium acetate | 48.4 | - | - | - |
| G5B5_04715 | *mtrE* | Triton X-100 | 48.4 | - | - | - |
| G5B5_08485 | *copA* | Cu, Ag | 48.4 | - | - | - |
| G5B5_15860 | *pgpA/ltpgpA* | As, Sb | 48.4 | - | - | - |
| G5B5_15165 | *sh-fabI* | Triclosan | 48.0 | sh-fabI | Enoyl-acyl-carrier-protein | 50.8 |
| G5B5_16145 | *cusA/ybdE* | Cu, Ag | 47.9 | *cusA/ybdE* | CusA/CzcA family heavy metal efflux RND transporter | 48.0 |
| G5B5_01825 | *copS* | Cu | 47.5 | - | - | - |
| G5B5_15680 | *mtrD* | Triton X-100 | 47.5 | - | - | - |
| G5B5_16090 | *mexY* | EtBr | 47.4 | - | - | - |
| G5B5_12645 | *soxS* | Zn | 47.2 | - | - | - |
| G5B5_12930 | *arsT* | As | 46.9 | *arsT* | Thioredoxin-disulfide reductase | 51.9 |
| G5B5_06355 | *modA* | W, Mo | 46.9 | - | - | - |
| G5B5_06715 | *oprN* | Triclosan | 46.9 | - | - | - |
| G5B5_07405 | *vmeC* | Sodium Glycocholate and others | 46.9 | *mexC* | MexC family multidrug efflux RND transporter subunit | 31.3 |
| G5B5_12535 | *pstA* | As | 46.7 | *pstA* | Phosphate transporter permease subunit PtsA | 47.2 |
| G5B5_04020 | *phoR* | BAC | 46.7 | - | - | - |
| G5B5_04280 | *oqxA* | BAC | 46.7 | - | - | - |
| G5B5_08775 | *ctpV* | Cu | 46.5 | - | - | - |
| G5B5_03175 | *tbtB* | TBT | 46.2 | - | - | - |
| G5B5_00865 | *ctpV* | Cu | 45.9 | - | - | - |
| G5B5_07315 | *copA* | Cu, Ag | 45.9 | - | - | - |
| G5B5_16340 | *ttgB* |  | 45.9 | - | - | - |
| G5B5_16800 | *copB* | Cu | 45.9 | - | - | - |
| G5B5_00800 | *arsH* | AS | 45.7 | - | - | - |
| G5B5_05205 | *bepE* | SDC | 45.7 | - | - | - |
| G5B5_08125 | *aioE* | As | 45.7 | *aioE* | NAD(P)/FAD-dependent oxidoreductase | 42.5 |
| G5B5_14315 | *czcS* | Cd, Zn, Co | 45.7 | - | - | - |
| G5B5_14920 | *czrA* | Zn, Cd | 45.7 | - | - | - |
| G5B5_10760 | *vmeB* | BAC | 45.7 | - | - | - |
| G5B5_11005 | *cpxR* | H2O2 | 45.7 | *cpxR* | DNA-binding response regulator | 46.3 |
| G5B5_00945 | *nikC* | Ni | 45.6 | *nikC* | Ni ABC transporter permease subunit NikC | 46.1 |
| G5B5_00440 | *chrR* | Cr, Fe, H2O2 | 45.5 | *yieF* | NAD(P)H-dependent oxidoreductase | 30.5 |
| G5B5_03750 | *mtrR* | Triton X-100 | 45.5 | - | - | - |
| G5B5_05605 | *actP/yjcG* | Sodium Glycocholate | 45.5 | - | - | - |
| G5B5_05665 | *fecE* | Ni, Co | 45.5 | - | - | - |
| G5B5_05745 | *mexX* | EtBr | 45.5 | - | - | - |
| G5B5_06575 | *fbpC* | Fe, Ga | 45.5 | - | - | - |
| G5B5_07095 | *nixA* | Ni | 45.5 | *nrsS* | Hypothetical protein | 30.4 |
| G5B5_11940 | *tbtB* | TBT | 45.2 | - | - | - |
| G5B5_09275 | *cnrH* | Co, Ni | 45.2 | *copB* | Cu-translocating P-type ATPase | 31.5 |
| G5B5_09475 | *smeE* | Triclosan | 45.2 | - | - | - |
| G5B5_12380 | *wtpB* | W, Mo | 45.2 | - | - | - |
| G5B5_02995 | - | - | - | *arsM* | Arsenite S-adenosylmethyltransferase | 60.0 |
| G5B5_05125 | - | - | - | *mtrD* | Phospho-N-acetylmuramoyl-pentapeptide-transferase | 57.6 |
| G5B5_10215 | - | - | - | *mntR* | Inner membrane protein YbiR | 57.2 |
| G5B5_09640 | - | - | - | *glpF* | Glycerol kinase | 56.9 |
| G5B5_03300 | *fbpC* | Fe, Ga | 32.6 | *ruvB* | DNA helicase RuvB | 56.7 |
| G5B5_04040 | - | - | - | *wtpA* | W ABC transporter substrate-binding protein WtpA | 53.3 |
| G5B5_01300 | *tbtA* | TBT | 33.8 | *zupT/ygiE* | Zn transporter ZupT | 51.1 |
| G5B5_16830 | - | - | - | *cmtR* | ArsR family transcriptional regulator | 50.0 |
| G5B5_08815 | *acrD* | Cu, Zn | 32.8 | *copA* | Cu-translocating P-type ATPase | 50.0 |
| G5B5_01765 | *nczA* | Ni, Co, Zn | 42.2 | *modC* | ABC-type spermidine/putrescine transport system | 50.0 |
| G5B5_08510 | *mreA* | Ni, Zn | 43.8 | *mreA* | Metal resistance protein | 50.0 |
| G5B5_16220 | - | - | - | *zupT/ygiE* | Zn transporter ZupT | 50.0 |
| G5B5_00905 | *hsmR* | EtBr | 36.4 | *arsT* | Thioredoxin-disulfide reductase | 49.0 |
| G5B5_11375 | *gadW/yhiW* | HCl | 42.4 | *mtrA* | AraC family transcriptional regulator | 48.8 |
| G5B5_08230 | *arsT* | As | 43.8 | *merA* | Mercury (II) reductase | 48.6 |
| G5B5_09395 | - | - | - | *vceA* | Efflux transporter periplasmic adaptor subunit | 48.4 |
| G5B5_11185 | - | - | - | *copS* | Sensor histidine kinase | 47.7 |
| G5B5_12325 | *ybtP* | Fe | 30.4 | *ziaA* | Ca-translocating P-type ATPase | 47.1 |
| G5B5_05120 | - | - | - | *aioE* | NAD(P)/FAD-dependent oxidoreductase | 46.2 |
| G5B5_04590 | *irlR* | Cd, Zn | 44.6 | *irlR* | DNA-binding response regulator | 46.2 |
| G5B5_03145 | *recG* | Cr, Te, Se | 33.3 | *mdtI/ydgE* | Multidrug/spermidine transporter subunit MdtI | 45.9 |
| G5B5_13015 | *adeB* | Pyronin Y | 40.0 | *adeB* | Multidrug efflux RND transporter permease subunit | 45.7 |
| G5B5_12855 | *bepE* | SDC | 44.4 | *bepE* | Multidrug efflux RND transporter permease subunit | 45.7 |
| G5B5_13845 | *vmeV* | SDS | 33.3 | *trgB* | Hypothetical protein | 45.6 |
| G5B5_12165 | *gadB* | HCl | 36.1 | *ctpV* | Metal cation transporter P-type ATPase CtpV | 45.6 |
| G5B5_04210 | *comR/ycfQ* | Cu | 43.2 | *comR/ycfQ* | TetR/AcrR family transcriptional regulator | 45.5 |
| G5B5_13340 | - | - | - | *copD* | Hypothetical protein | 45.5 |
| G5B5_14950 | - | - | - | *pbrT* | Fe permease | 45.5 |
| G5B5_15200 | - | - | - | *pcm* | Protein-L-isoaspartate O-methyltransferase | 45.5 |
| G5B5_12960 | *mexF* | Triclosan | 31.7 | *fecD* | Fe-dicitrate transporter subunit FecD | 45.2 |

| Table S5: Significantly upregulated proteins in the proteomic profile of *Granulicella* sp 5B5 with the addition of Mn with hits against BacMet gene databases. Annotation performed against the experimentally confirmed and predicted resistance genes databases using BacMet Scan software. Only genes with hits ≥ 40% identity in one of the databases are shown. | | | | | | |
| --- | --- | --- | --- | --- | --- | --- |
| ORF | EXP | Compound | Iden | PRE | Compound | Iden |
| G5B5_06575 | *fbpC* | Fe, Ga | 45.5 | - | - | - |
| G5B5_05650 | *cueA* | Cu, Ag | 43.5 | *cueA* | Cu-translocating P-type ATPase | 39.3 |
| G5B5_00055 | *chrA* | Cr | 38.7 | - | - | - |
| G5B5_01745 | *fbpB* | Fe, Ga | 38.3 | - | - | - |
| G5B5_06295 | *aioR/aoxR* | As | 36.7 | - | - | - |
| G5B5_03725 | *pmrC* | Fe | 36.4 | *mtrA* | AraC family transcriptional regulator | 32.7 |
| G5B5_03225 | *gadB* | HCl | 35.9 | *copA* | Cu-translocating P-type ATPase | 30.9 |
| G5B5_01965 | *mexF* | Triclosan | 34.7 | - | - | - |
| G5B5_03160 | *bepG* | SDC | 34.3 | - | - | - |
| G5B5_06075 | *nczA* | Ni, Co, Zn | 34.1 | - | - | - |
| G5B5_08550 | *actR* | Cd, Zn, HCl | 33.8 | *zraR/hydH* | Fis family transcriptional regulator | 30.0 |
| G5B5_06495 | *tcrA* | Cu | 33.3 | *copA* | Cu-translocating P-type ATPase | 39.6 |
| G5B5_11930 | *mexY* | EtBr | 33.3 | - | - | - |
| G5B5_07390 | *bcrA* | BAC | 33.0 | *bcrA* | TetR/AcrR family transcriptional regulator | 33.0 |
| G5B5_13190 | *adeT1* | BAC | 32.6 | - | - | - |
| G5B5_13570 | *copR* | Cu | 32.5 | *corR* | Fis family transcriptional regulator | 30.4 |
| G5B5_09210 | *ibpB* | H2O2 | 32.0 | *ibpB* | Heat shock chaperone IbpB | 36.7 |
| G5B5_02980 | *fabL/ygaA* | Triclosan | 32.0 | *fabL/ygaA* | Enoyl-acyl-carrier-protein | 31.7 |
| G5B5_02655 | *copA* | Copper (Cu), Silver (Ag) | 31.9 | *yieF* | NAD(P)H-dependent oxidoreductase | 32.1 |
| G5B5_00640 | *mtrD* | Triton X-100 | 31.4 | - | - | - |
| G5B5_03020 | *farR* | Palmitic acid | 31.3 | *farR* | Homoprotocatechuate degradation operon regulator HpaR | 30.6 |
| G5B5_15040 | *mexK* | Triclosan | 31.1 | *arsM* | As S-adenosylmethyltransferase | 33.3 |
| G5B5_11390 | *fabK* | Triclosan | 30.8 | *fabK* | Enoyl-acyl-carrier-protein | 33.3 |
| G5B5_04810 | *copB* | Cu | 30.2 | - | - | - |
| G5B5_14875 | *troB* | Zn, Mn, Fe | 30.0 | *troB* | Metal ABC transporter ATP-binding protein | 31.2 |
| G5B5_08555 | - | - | - | *fabL/ygaA* | Enoyl-acyl-carrier-protein | 36.8 |
| G5B5_13250 | - | - | - | *chrB* | Hypothetical protein | 32.2 |
| G5B5_12545 | - | - | - | *pstA* | PhoU and BPD transp 1 and ABC tran domain containing protein | 30.7 |
