# Supplementary material for: Impact of Different Trace Elements on the Growth and Proteome of Two Strains of Granulicella, Class “Acidobacteriia”
Source: Front Microbiol. 2020 Jun 18;11:1227. doi: 10.3389/fmicb.2020.01227 (PMC7315648; doi:10.3389/fmicb.2020.01227)

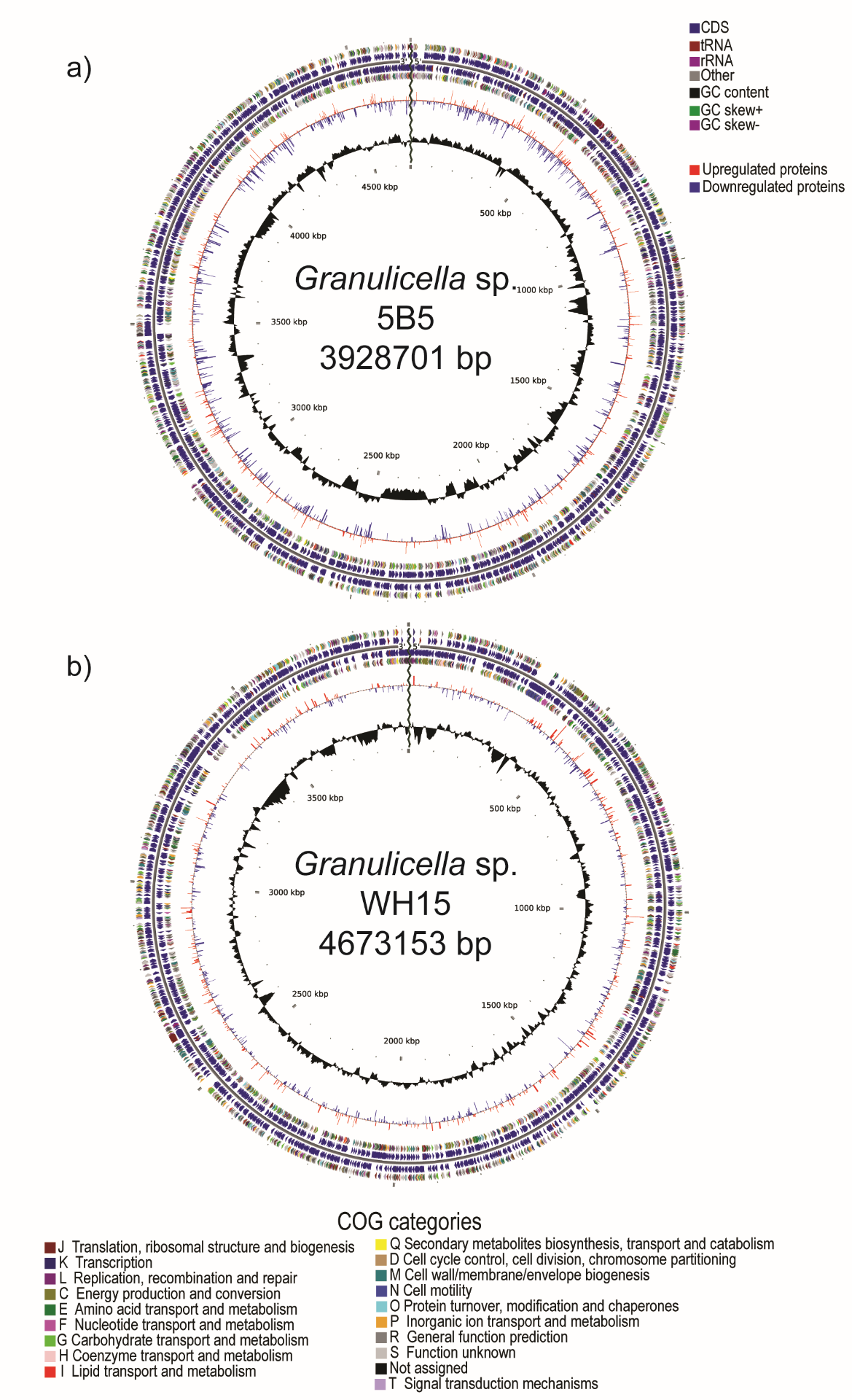


Figure S1: Graphical circular genome map of *Granulicella* sp. a) 5B5 and b) WH15. Rings from the outside to the inside indicate COG categories, coding sequences, upregulated (red) and downregulated (blue) proteins upon the addition of Mn to bacterial cultures and GC content.

Figure S2: a) Dendogram showing the relationships between *Granulicella* species based on comparisons between 16S rRNA gene sequences. The dendogram was constructed by using maximum-likelihood (Tamura-Nei model) analysis. Bootstrap values (expressed as percentages of 1,000 replications) are shown at branch points. *Burkholderia glathei* was used as outgroup. The strains used in this work are highlighted in red bold letters. b) Genome-wide comparison of *Granulicella* strains 5B5 and WH15. Linear chromosomal maps were build using AliTV v. 1.0 visualization software, based on whole-genome alignments with Lastz v. 1.0.4 aligner. The picture depicts a pairwise comparison, expressed as percentage of nucleotide similarity, that connect different homologous genomic regions. Genomes are completely finished and pictured in blue.


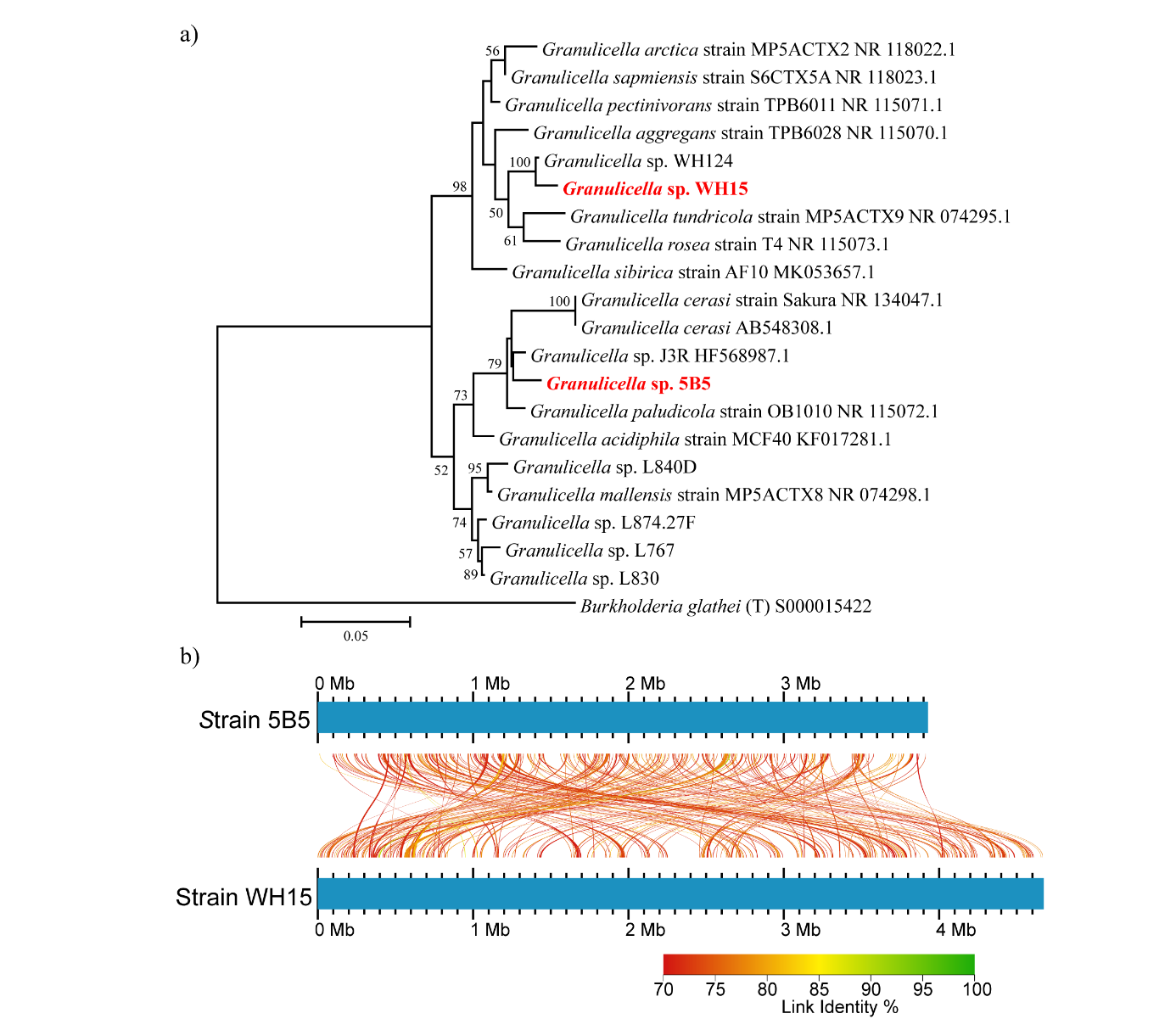


Figure S3: Growth curves of *Granulicella* sp. a) 5B5 and b) WH15 in PSYL 5 liquid culture medium with addition of Mn and control without addition of metal. The arrow indicates the timepoint when samples were collected for proteomics analysis. The error bar is the standard error of the mean and indicates differences in response variable between different treatments.


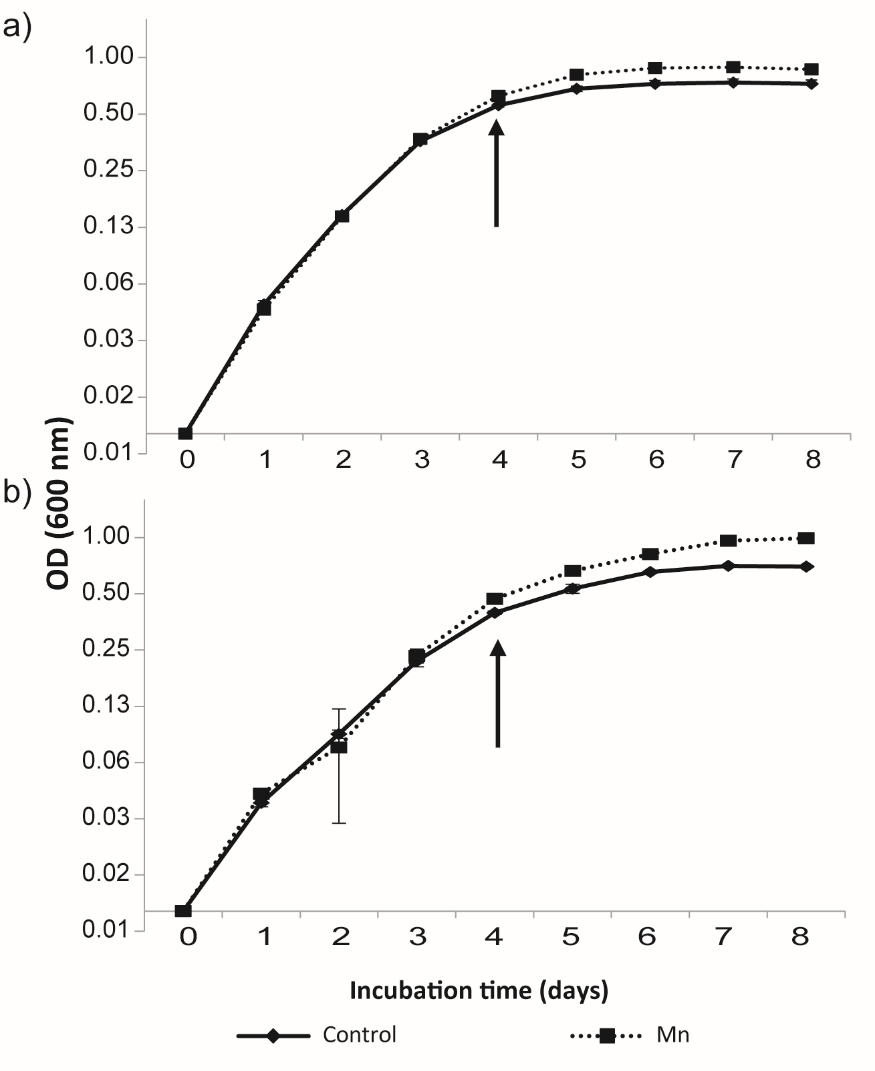


Figure S4: Expression pattern of proteins under control and manganese treatment of *Granulicella* sp. a) 5B5) and b) WH15. All proteins identified in at least two out of three replicates (excluding on/off proteins). Log_2_ fold change is indicated by a red line.


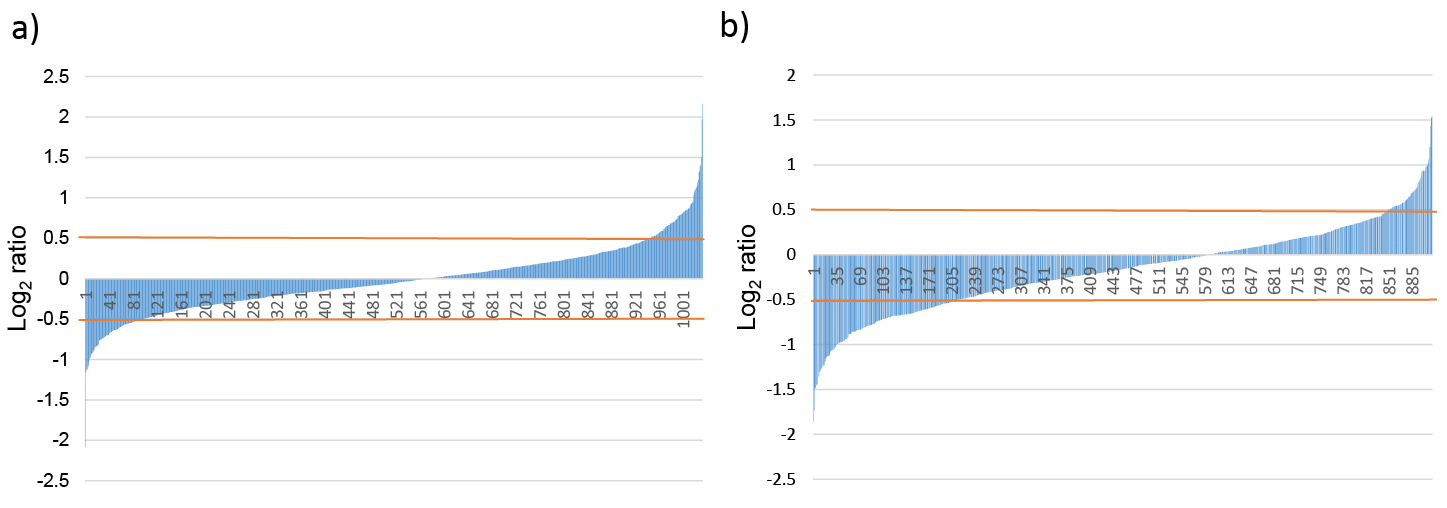


Figure S5: Voronoi treemap visualization of protein expression patterns of *Granulicella* sp. a) 5B5 and b) WH15 spectrum under control and manganese treatments. Functional classification was done using Prophane 2.0 (www.prophane.de) and is based on eggnog database, B function level “subrole”. Each cell represents a quantified protein; proteins are clustered according to their function. Proteins with higher amount under control conditions (no metal) are depicted in blue, proteins with higher amount in manganese treatment are depicted in red.


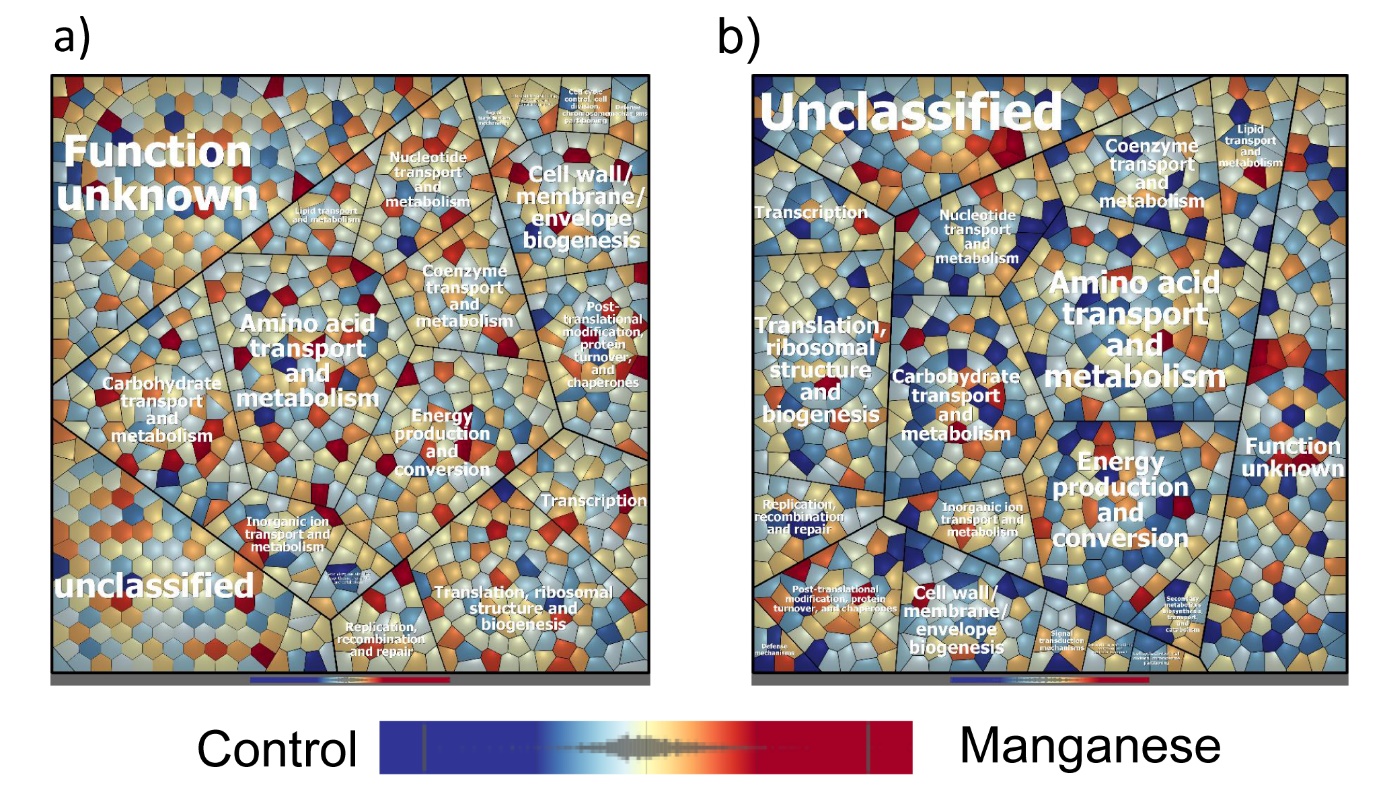

Supplement: Supplementary file 2 [file Data_Sheet_2.docx]
